# Supplementary material for: Allosteric binders of ACE2 are promising anti-SARS-CoV-2 agents
Source: bioRxiv. 2022 Mar 16:2022.03.15.484484. Preprint. [Version 1] doi: 10.1101/2022.03.15.484484 (PMC8936107; doi:10.1101/2022.03.15.484484)
Supplement: Supplement 1 [file media-1.pdf]

Allosteric binders of ACE2 are promising anti-SARS-CoV-2 agents

Joshua E. Hochuli[#1,2], Sankalp Jain[#3], Cleber Melo-Filho[1], Zoe L. Sessions[1], Tesia Bobrowski[1], Jun Choe[3], Johnny Zheng[3], Richard Eastman[3], Daniel C. Talley[3], Ganesha Rai[3], Anton Simeonov[3], Alexander Tropsha[1*], Eugene N. Muratov[1*], Bolormaa Baljinnyam[3*], Alexey V. Zakharov[3*].

1. Molecular Modeling Laboratory, UNC Eshelman School of Pharmacy, University of North Carolina, Chapel Hill, NC.

2. Curriculum in Bioinformatics and Computational Biology, University of North Carolina, Chapel Hill, NC.

3. National Center for Advancing Translational Sciences, National Institutes of Health, Rockville, MD.

[#] These authors contributed equally.

* Corresponding authors

Supplementary Information

Supplementary Table 1: Ligand-based pharmacophore models generated for screening (LBP).

Pharmacophore	pharmacophore type	cluster distance
H-HBA-HBA-HBD-HBD-NI-XV44	MFP	0.4
AR-AR-AR-H-HBA-HBA-HBA-HBD-HBD-HBD-XV46	MFP	0.4
AR-AR-H-H-HBA-HBA-HBA-HBA-HBD-XV42	MFP	0.4
H-HBA-HBA-HBA-HBA-HBD-HBD-XV34	MFP	0.4
HBA-HBA-HBA-HBA-HBA-HBD-HBD-HBD-XV37	MFP	0.4
AR-AR-AR-H-HBA-HBA-HBA-HBD-HBD-HBD-XV46	MFP	0.6
H-HBA-HBA-HBA-NI-XV42	MFP	0.6
HBA-HBA-HBA-XV27	MFP	0.6
AR-AR-H-H-HBA-HBA-HBA-HBD-XV35	MFP	0.6
AR-AR-AR-HBA-HBA-HBA-HBA-HBA-HBD-XV47	MFP	0.6
AR-H-HBA-HBA-HBA-HBA-HBD-HBD-HBD-XV41	MFP	0.6
AR-HBA-HBA-HBA-HBD-XV37	MFP	0.6

HBA-HBA-HBA-HBA-HBA-HBA-HBD-PI-XV40	MFP	0.6
AR-AR-H-HBD-XV29	MFP	0.6
HBA-HBD-HBD-HBD-XV27	MFP	0.6
HBA-HBA-HBD-XV26	MFP	0.6
HBA-HBA-HBA-XV19	MFP	0.6
H-HBA-HBA-XV21	MFP	0.6
AR-HBA-HBA-HBA-XV38	MFP	0.6
AR-AR-H-HBA-HBA-HBD-HBD-NI-XV37	MFP	0.6
AR-H-HBA-HBA-HBD-HBD-XV29	MFP	0.7
AR-AR-H-HBD-XV30	MFP	0.7
AR-H-HBA-HBA-HBA-HBD-NI-XV36	MFP	0.7
AR-HBA-HBA-HBD-XV21	MFP	0.7
AR-H-HBA-HBA-XV13	MFP	0.7
HBA-HBA-HBD-HBD-XV21	MFP	0.7
HBA-HBA-HBD-XV18	MFP	0.7
AR-H-HBA-HBA-HBA-HBD-XV34	MFP	0.7
HBA-HBA-HBA-XV28	MFP	0.7
H-H-HBA-HBA-NI-XV33	MFP	0.7
HBA-HBA-HBA-HBD-XV22	MFP	0.7
HBA-HBA-HBD-HBD-HBD-HBD-XV39	MFP	0.7
H-HBA-HBA-HBA-HBD-XV31	MFP	0.7
AR-H-HBA-HBD-XV24	MFP	0.8
HBA-HBA-HBA-XV18	MFP	0.8
HBA-HBA-HBA-HBD-XV1	MFP	0.8
H-HBA-HBA-XV31	SFP	0.4
AR-AR-AR-H-HBA-HBA-HBA-XV34	SFP	0.4
AR-AR-AR-H-XV17	SFP	0.4
AR-HBA-HBA-XV25	SFP	0.4
AR-AR-HBA-HBA-HBD-XV24	SFP	0.4
H-HBA-HBA-HBA-HBD-XV32	SFP	0.4
AR-AR-H-H-H-XBD-XV31	SFP	0.4
HBA-HBA-HBA-HBA-HBD-HBD-XV35	SFP	0.4
AR-AR-AR-H-HBA-HBA-HBA-XV34	SFP	0.6
H-HBA-HBA-NI-XV36	SFP	0.6
AR-HBA-HBA-XV25	SFP	0.6
AR-H-H-HBA-XV30	SFP	0.6
AR-H-HBA-HBA-HBD-XV33	SFP	0.6
AR-H-HBA-HBA-HBD-HBD-XV36	SFP	0.6

AR-H-HBA-HBD-XV32	SFP	0.6
HBA-HBA-HBA-HBD-PI-XV35	SFP	0.6
HBA-HBA-HBA-NI-XV24	SFP	0.6
AR-AR-H-XV13	SFP	0.6
AR-H-HBA-XV17	SFP	0.6
H-HBA-HBA-HBD-XV29	SFP	0.6
HBA-HBA-HBD-XV21	SFP	0.6
AR-H-HBA-XV22	SFP	0.6
AR-HBA-HBA-XV22	SFP	0.6
HBA-HBA-HBD-XV20	SFP	0.6
HBA-HBA-HBA-XV16	SFP	0.6
AR-HBA-HBA-XV28	SFP	0.6
AR-AR-H-HBA-XV27	SFP	0.6
H-HBD-HBD-XV30	SFP	0.7
AR-AR-H-XV19	SFP	0.7
HBA-HBA-HBD-NI-XV17	SFP	0.7
H-HBA-HBA-HBD-HBD-XV28	SFP	0.7
AR-H-H-HBA-XV31	SFP	0.7
HBA-HBA-HBD-XV20	SFP	0.7
HBA-HBA-HBD-XV21	SFP	0.7
H-HBA-HBA-HBD-XV26	SFP	0.7
AR-H-HBA-HBD-XV28	SFP	0.7
HBA-HBD-HBD-XV27	SFP	0.7
H-HBA-HBA-XV22	SFP	0.7
HBA-HBA-NI-XV18	SFP	0.7
H-HBA-HBA-XV28	SFP	0.7
HBA-HBA-HBA-HBA-HBD-XV37	SFP	0.7
H-HBA-HBA-XV29	SFP	0.7
H-HBA-HBA-XV29	SFP	0.7
HBA-HBA-HBA-XV23	SFP	0.8
HBA-HBA-HBD-XV16	SFP	0.8
AR-HBA-HBD-XV8	SFP	0.8
H-H-HBA-XV24	SFP	0.8
AR-HBA-HBA-XV21	SFP	0.8
AR-HBA-HBA-XV11	SFP	0.8
H-HBA-HBA-XV18	SFP	0.8
H-H-HBA-XV39	SFP	0.8
AR-AR-H-XV17	SFP	0.8
HBA-HBA-HBD-XV4	SFP	0.8

MEP	merged-features pharmacophore
SFP	shared-features pharmacophore
H	Hydrophobic
HBA	H-Bond Acceptor
HBD	H-Bond Donor
PI	Positive Ionizable
NI	Negative Ionizable
AR	Aromatic Ring
XV	Exclusion Volume

(green box)	pharmacophore models that hit the majority (>20%) of active vs. inactive

Supplementary Table 2: Validation statistics for UNC models. Reported statistics are an average across each of five test folds. Final selected models are shown in **bold.** Acronyms: Correct Classification Rate (CCR), Positive Predictive Value (PPV), Negative Predictive Value (NPV).

**Model Type**	**Descriptor**	**CCR**	**PPV**	**NPV**
**Neural Network**	**Simplex**	**0.514**	**0.096**	**0.964**
Neural Network	RDKit	0.500	0	0.963
Neural Network	Morgan	0.500	0	0.963
**Gradient Boosting**	**Simplex**	**0.516**	**0.123**	**0.965**
**Gradient Boosting**	**RDKit**	**0.511**	**0.102**	**0.964**
Gradient Boosting	Morgan	0.495	0	0.963
**MuDRA**	**MuDRA**	**0.522**	**0.129**	**0.965**

Supplementary Table 3: Model performance of NCATS Stratified bagging models on the training and test dataset. Acronyms: Matthews correlation coefficient (MCC), Positive Predictive Value (PPV), Negative Predictive Value (NPV), Area under the receiver operating characteristic curve (AUC).

**Training set**									
**Descriptor**	**Sensitivity**	**Specificity**	**Accuracy**	**Balanced Accuracy**	**MCC**	**Precision/PPV**	**NPV**	**Recall**	**AUC**
**RDKit**	0.48	0.78	0.76	0.63	0.11	0.07	0.98	0.48	0.69
**Morgan**	0.30	0.87	0.85	0.58	0.09	0.08	0.97	0.30	0.69
**Avalon**	0.46	0.82	0.81	0.64	0.13	0.09	0.98	0.46	0.68
**Consensus**	0.38	0.85	0.83	0.61	0.12	0.09	0.97	0.38	0.72
**Test set**									
**RDKit**	0.41	0.83	0.81	0.62	0.11	0.08	0.97	0.41	0.62
**Morgan**	0.32	0.92	0.90	0.62	0.16	0.14	0.97	0.32	0.70
**Avalon**	0.27	0.87	0.85	0.57	0.08	0.07	0.97	0.27	0.57
**Consensus**	0.32	0.88	0.86	0.60	0.11	0.09	0.97	0.32	0.64

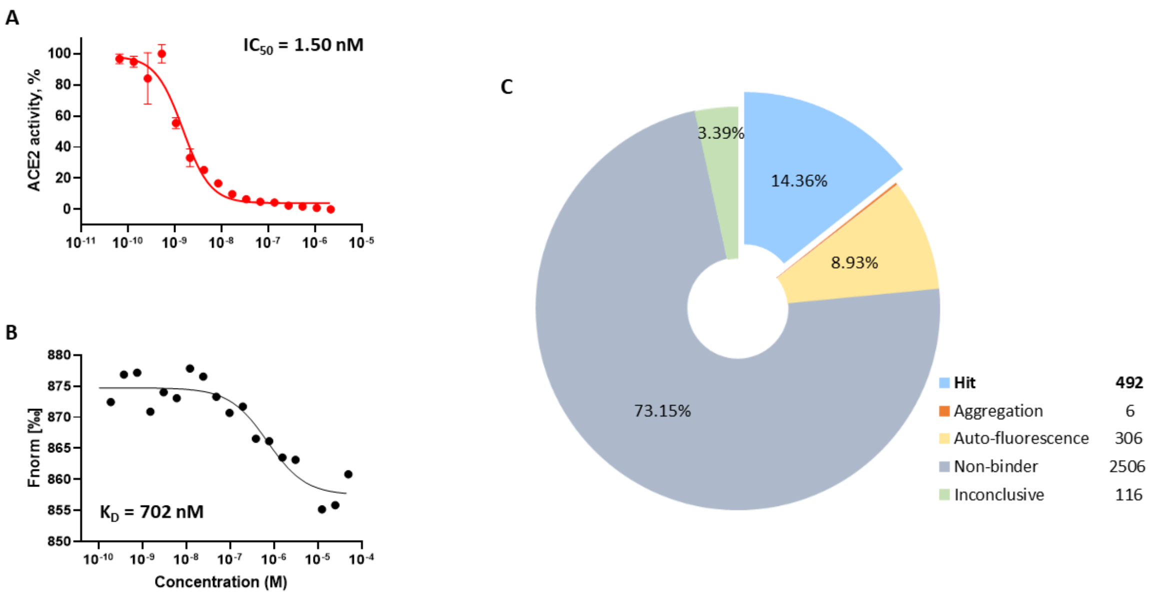

Figure S1

Supplementary Figure 1: Known ACE2 inhibitor MLN-4760 was tested in ACE2 screening assays to use as the control compound during screening. (A) Dose-response curve of MLN-4760 in ACE2 enzymatic assay with IC_{50} of 1.5 nM, (B) MNL-4760 binding to ACE2 measured by MST. (C) Summary of the small molecule library screening for ACE2 binders using MST grouped by compound categories with total number of compounds in each group.

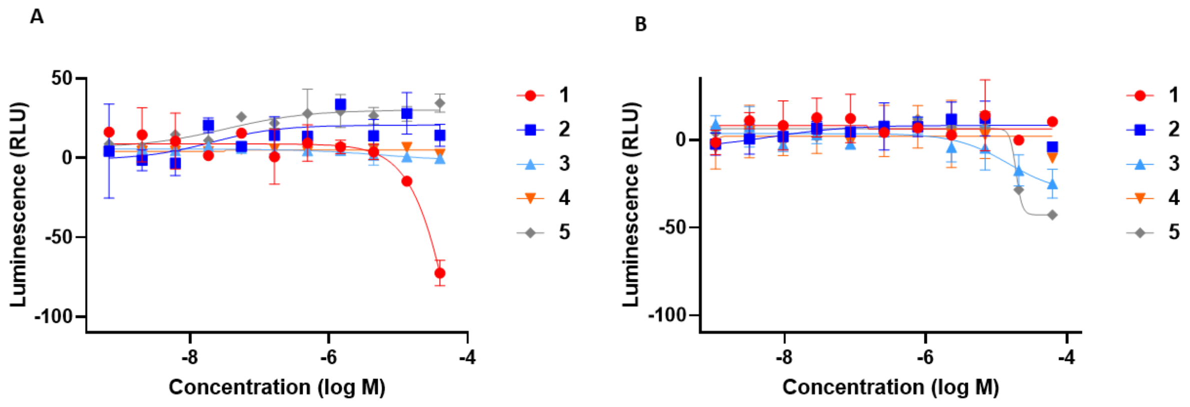

Supplementary Figure 2: Performance of the five ACE2 binders which showed inhibitory activity in the SARS-CoV-2 Fluc assay in the counterscreens: (A) cytotoxicity assay in A549-ACE2 cells, (B) firefly luciferase enzymatic assay.
